# Tricuspid mechanical valve replacement for severe tricuspid stenosis in a child who underwent surgical correction of complete atrioventricular septal defect, a rare long-term complications

**DOI:** 10.1186/s13019-024-02580-7

**Published:** 2024-02-09

**Authors:** Qi-Liang Zhang, Yu-Kun Chen, Shi-Hao Lin, Xin-Wei Du, Qiang Chen, Shun-Min Wang, Hua Cao

**Affiliations:** 1 Department of Cardiac Surgery, Fujian Children’s Hospital (Fujian Branch of Shanghai Children’s Medical Center), College of Clinical Medicine for Obstetrics & Gynecology and Pediatrics, Fujian Medical University, Fuzhou, China; 2 Department of Cardiac and Thoracic Surgery, Shanghai Children’s Medical Center, Shanghai Jiaotong University School of Medicine, Shanghai, China

**Keywords:** Tricuspid stenosis, Tricuspid valve replacement, Glenn, Surgery after complete atrioventricular septal defect

## Abstract

**Background:**

Complete atrioventricular septal defect is a complicated congenital heart malformations, and surgical correction is the best treatment, the severe tricuspid stenosis is a rare long-term complication after the surgery.

**Case presentation:**

We report a case with the complication of severe tricuspid stenosis 7 years after the surgical correction of complete atrioventricular septal defect in a child. Then the patient underwent tricuspid mechanical valve replacement, Glenn, atrial septostomy, and circumconstriction of the right pulmonary artery.

**Conclusions:**

The patient recovered successfully with good short-term.

## Introduction

Complete atrioventricular septal defects are complicated congenital heart malformations, including ostium primum atrial septal defects, inlet ventricular septal defects and common atrioventricular valves [1]. This defect occurs in 3 of 1000 live births and accounts for 7% of all congenital heart diseases [2]. Due to the presence of a large number of cardiac left-to-right shunts and valve regurgitation, respiratory tract infection and heart failure recurred. Therefore, they often need early surgical treatment [3]. With advances in surgical technique and improved understanding of atrioventricular valve morphology through echocardiography, outcomes for isolated atrioventricular septal defect have dramatically improved in the past few decades [4]. However, despite improved survival, several single-center studies have reported that up to 10% of patients, after initial complete atrioventricular septal defect repair, may require reoperation within 10 to 15 years to address valve regurgitation [5, 6]. Furthermore, the need for reoperation affects late survival after complete atrioventricular septal defect repair. Postoperative mitral regurgitation, tricuspid regurgitation and atrioventricular septal residual shunt are common complications [4]. However, severe tricuspid stenosis is a rare long-term complication that presents after the surgical correction of complete atrioventricular septal defects in children. We report a case of severe tricuspid stenosis that presented 7 years after surgical correction of a complete atrioventricular septal defect in a child and the treatment experience.

## Case report

**Fig. 1:**
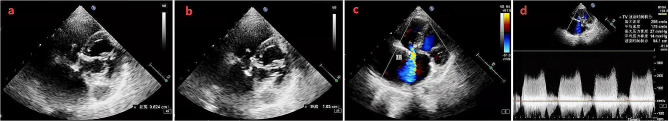
Result of echocardiography. **a**: Tricuspid valve stenosis was severe with valve opening distance of 0.64 cm. **b**: Tricuspid annulus was small with diameter of 1.03 cm. **c**: The tricuspid regurgitation was moderate to severe, with regurgitation velocity of 201 cm/s and pressure of 16 mmHg. **d**: The flow velocity of tricuspid valve was 258 cm/s, and peak pressure gradient was 27 mmHg

**Fig. 2:**
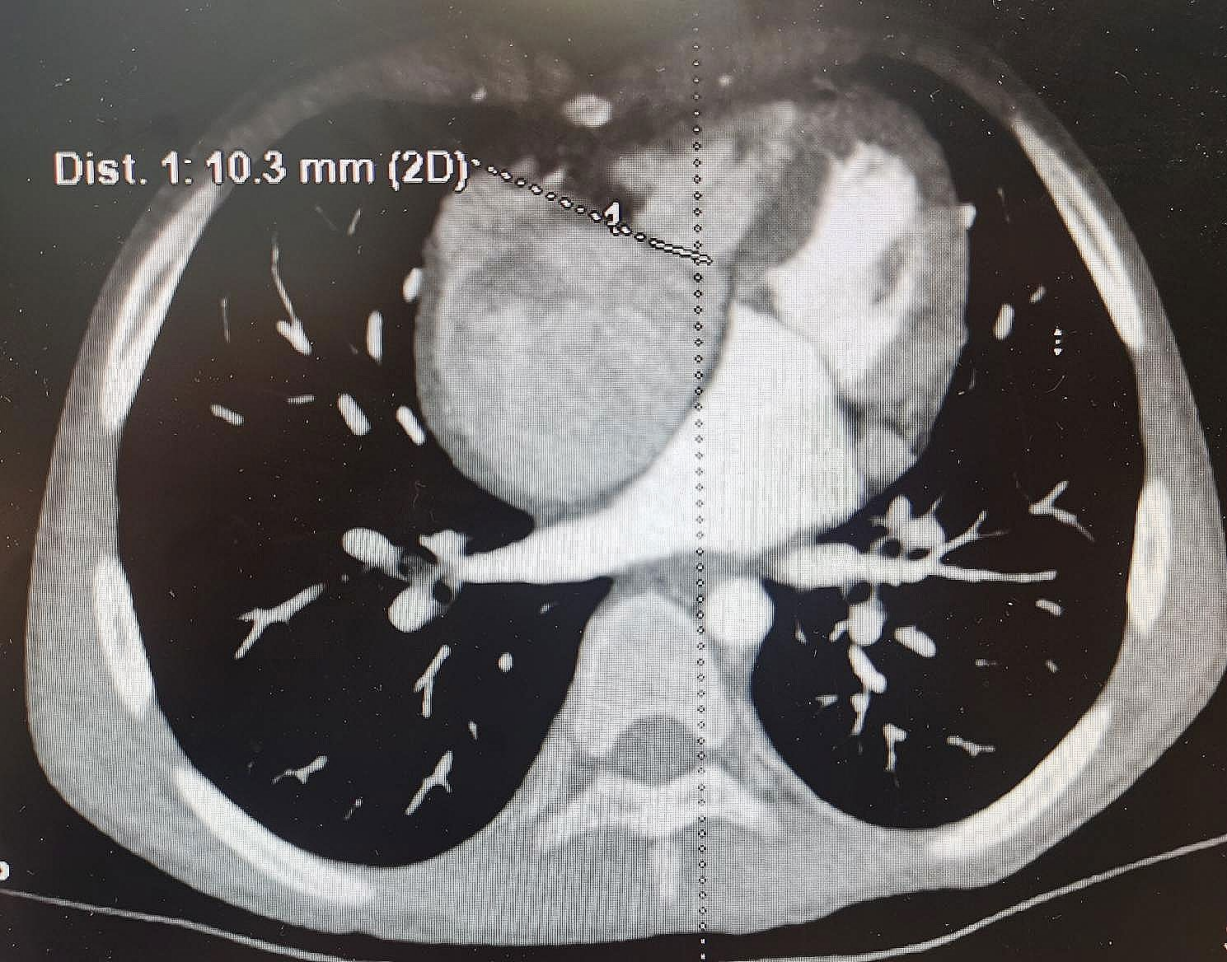
CT cardiac angiography showed that the right atrium was enlarged, the right ventricle was small, the diameter of Tricuspid annulus was 1.03 cm

A pediatric patient who was male, 7.5 years old, 19 kg, and 133 cm in height underwent surgical correction of a complete atrioventricular septal defect 7 years ago in another hospital. Postoperative echocardiography showed that there was no residual shunt in the atrial or ventricular septum and no mild mitral or tricuspid valve regurgitation. After discharge, the patient was not regularly followed up and was reexamined by echocardiography. Symptoms of shortness of breath after activity appeared 3 months ago. Symptoms of shortness of breath after activity worsened, combined with cough and sputum 1 week prior. There was no fever, cyanosis, chest pain, facial edema, abdominal distension, or other symptoms. Physical examination revealed a few rales in both lungs, normal rhythm, no murmur in the precordial area, no distension of the jugular veins, and no edema in either lower limb. Echocardiography showed that the right atrium was significantly enlarged, and the right ventricle was small. Tricuspid valve stenosis was severe. The tricuspid annulus was small, with a diameter of 1.03 cm and a valve opening distance of 0.64 cm. The flow velocity of the tricuspid valve was 258 cm/s, and the peak pressure gradient was 27 mmHg. The tricuspid regurgitation was moderate to severe, with a regurgitation velocity of 201 cm/s and pressure of 16 mmHg. The estimated pulmonary artery pressure was 26 mmHg. Mitral regurgitation was mild, the diameter of the mitral ring was 1.88 cm, and the valve orifice velocity was 101 cm/s. The Z score of the tricuspid annulus was − 3.78, and the Z score of the mitral annulus was 0.227 (Fig. 1). Cardiac CT angiography showed that the right atrium was enlarged, the right ventricle was small, and the diameter of the tricuspid annulus was 1.03 cm (Fig. 2). The results of cardiac catheterization also showed that the right atrium was significantly enlarged, the right ventricle was small, the ring of the tricuspid valve was narrow, and the atrial septum and ventricular septum were intact. The pulmonary trunk and the left and right pulmonary arteries were well developed, and the pulmonary small vessels were also well developed. The pulmonary capillary wedge pressure was 16/10 mmHg with an average pressure of 12 mmHg. The pulmonary vascular resistance index was 1.21. Abdominal color Doppler ultrasound showed hepatomegaly. According to the clinical manifestations and auxiliary examination results, the diagnosis was as follows: severe tricuspid valve stenosis with moderate to severe regurgitation, mild mitral valve regurgitation, cardiac function insufficiency, right ventricular dysplasia, respiratory tract infection, and surgical correction of complete atrioventricular septal defect. According to the patient’s condition, the surgical protocol included tricuspid mechanical valve replacement, Glenn procedures, atrial septostomy and right pulmonary artery banding.

### Surgical techniques

**Fig. 3:**
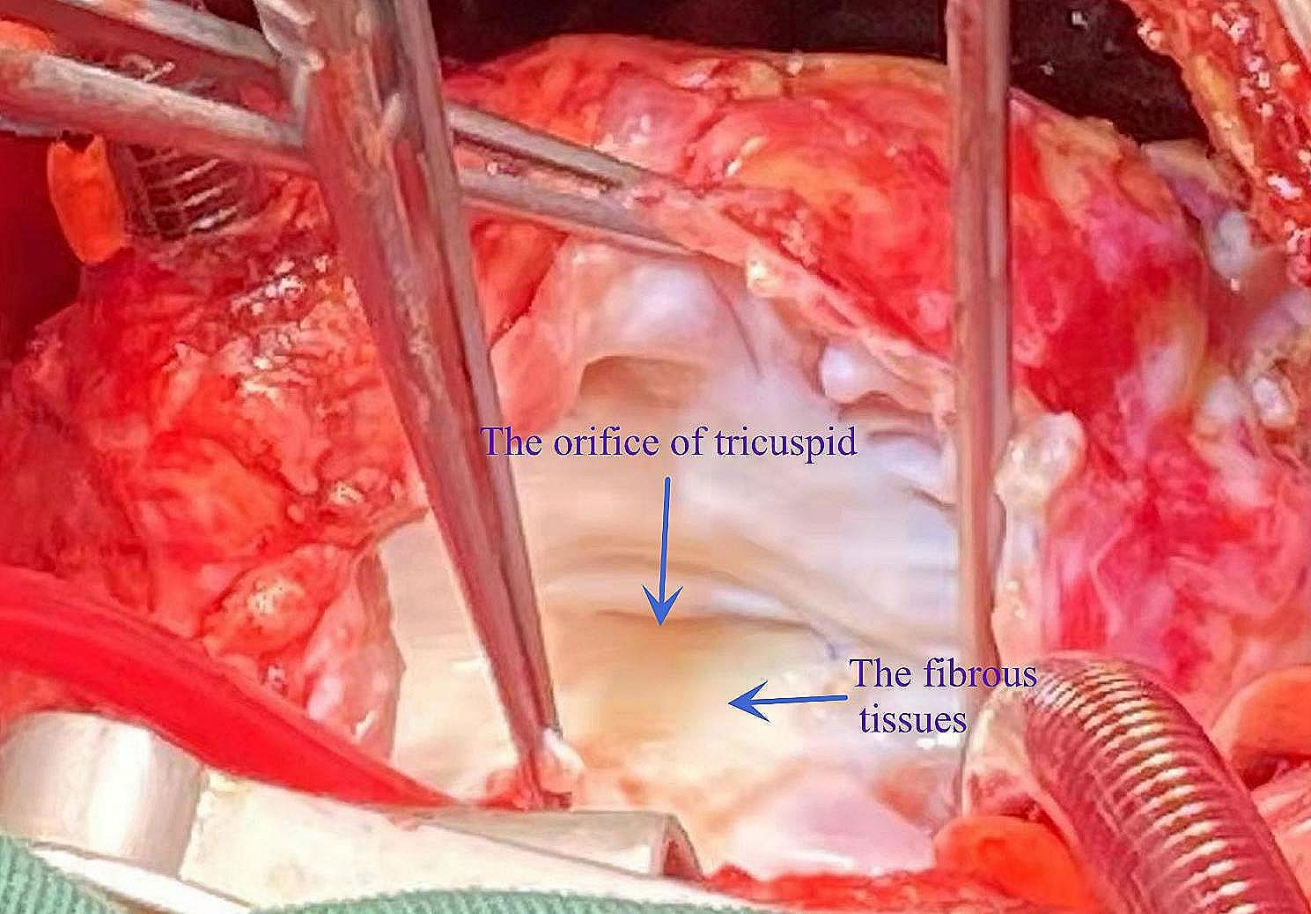
Tricuspid valve was dysplasia, fibrous tissue was hyperplasia around the tricuspid valve ring and valve leaflet contracture which lead to severe tricuspid valve stenosis and insufficiency

**Fig. 4:**
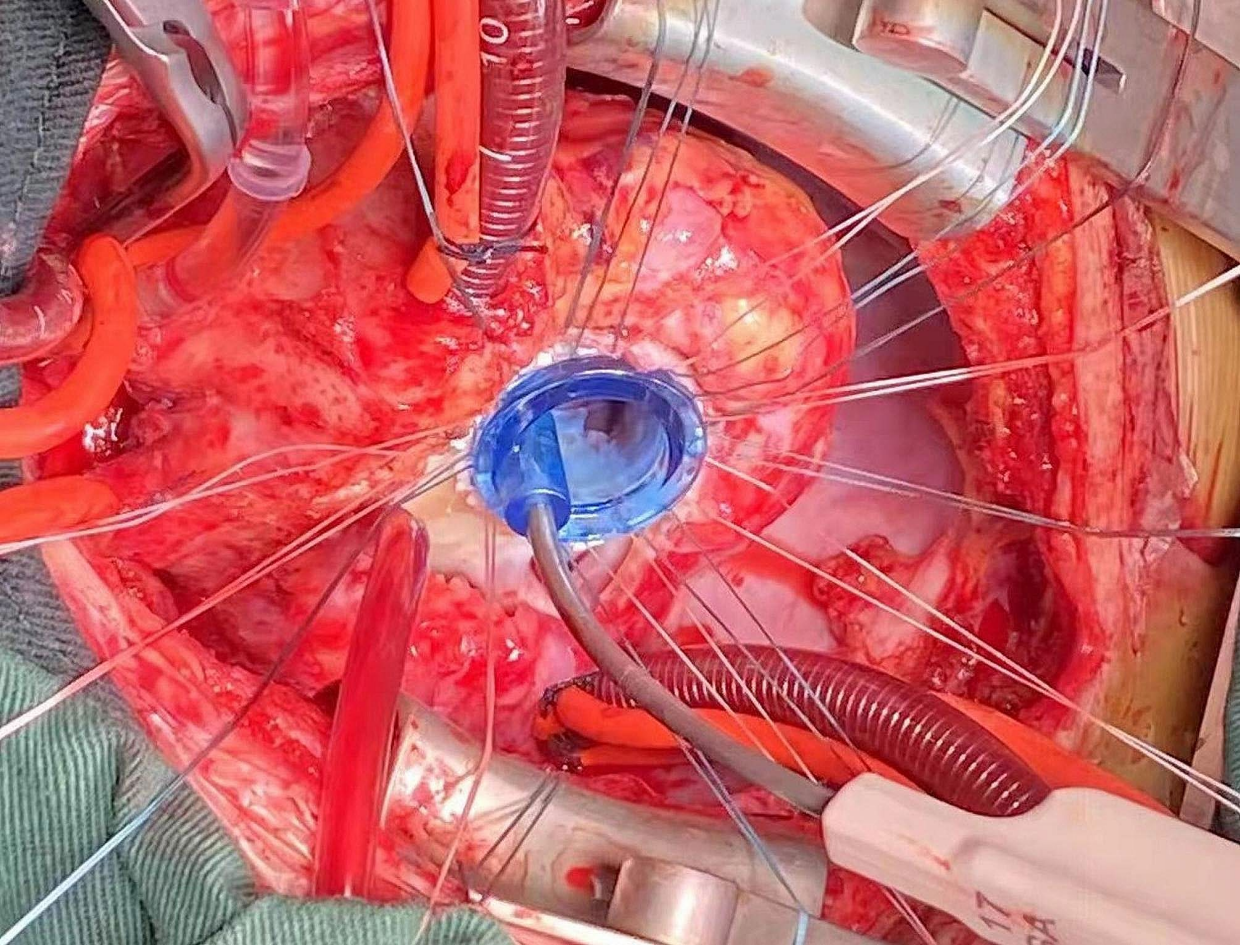
All the tricuspid leaves and subvalvular tissues were removed, and only the No. 17 mm valve tester could be passed

**Fig. 5:**
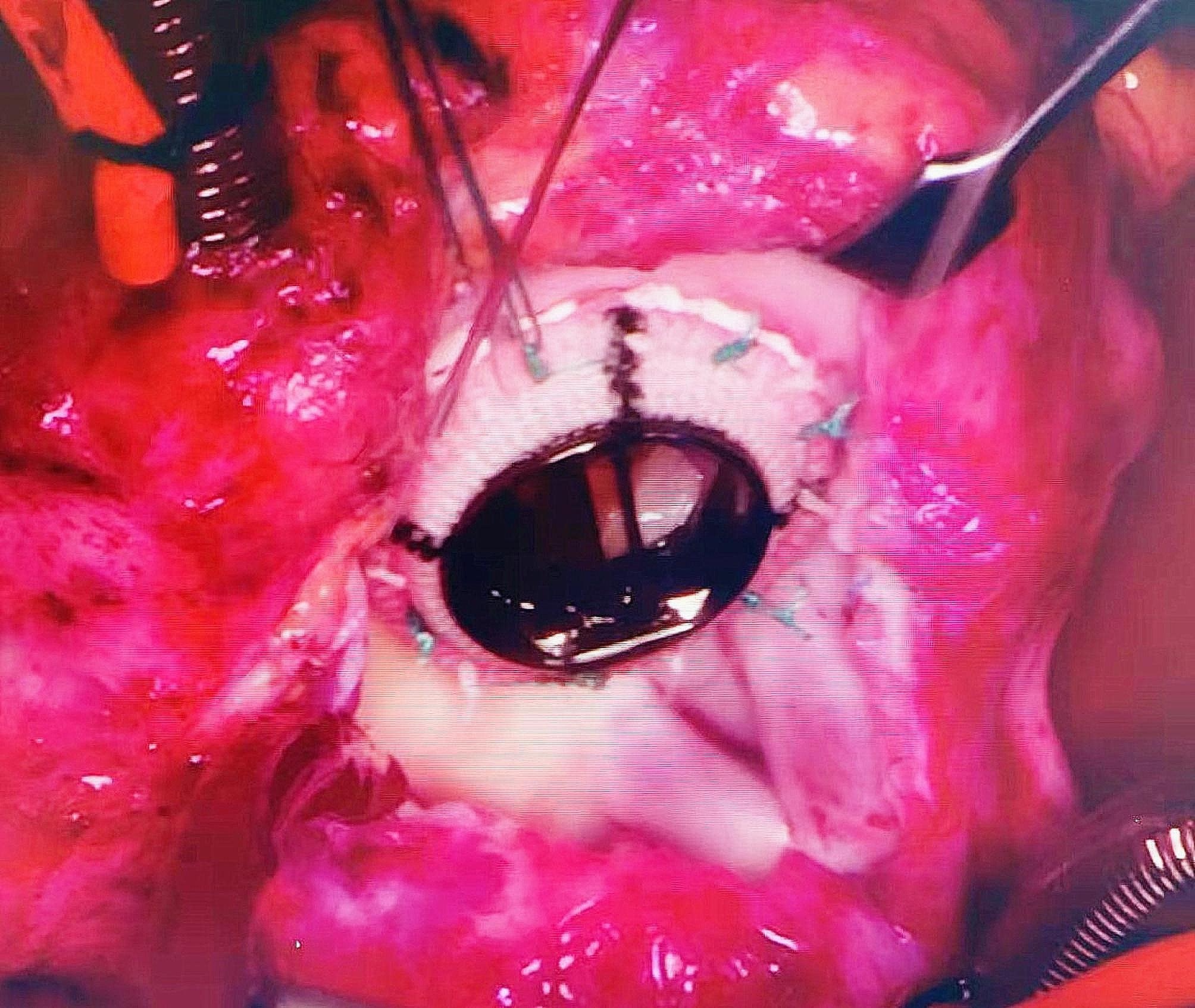
Sixteen stitches were sutured along the valve ring and the No. 16 mm Metronic mitral valve was implanted

**Fig. 6:**
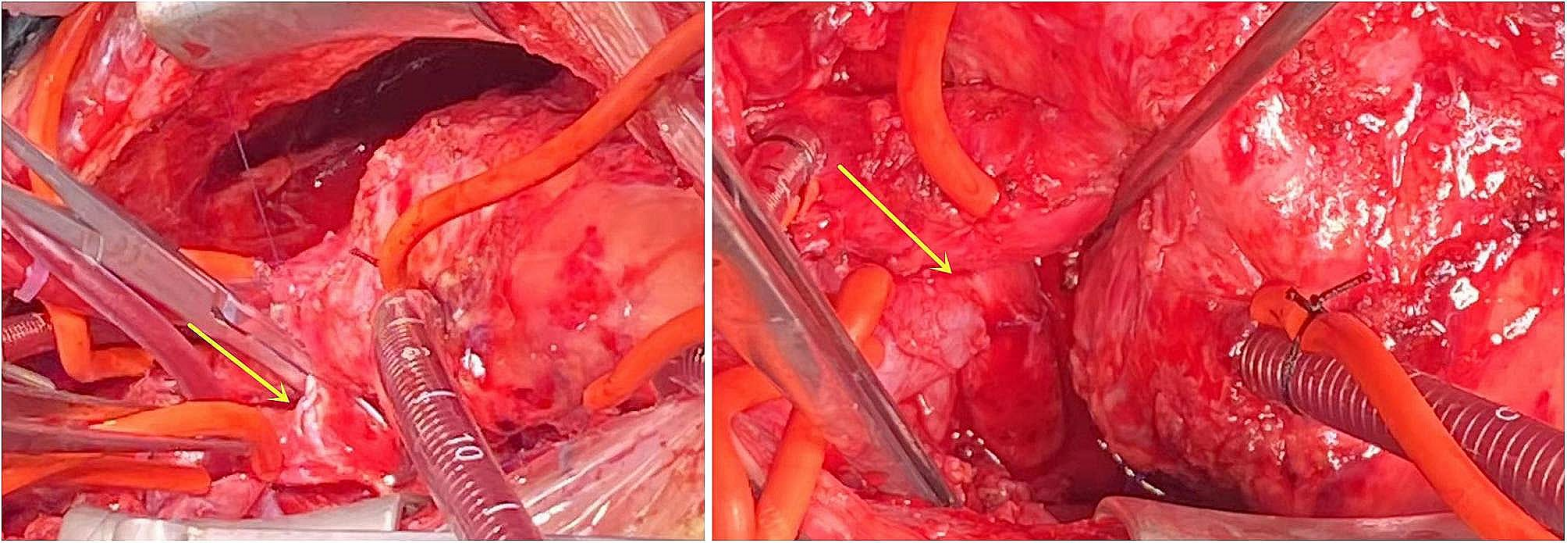
The Glenn procedure was performed

After general anesthesia and median resternotomy, extracorporeal circulation was established using ascending aorta and bicaval cannulation. The right atrium was opened after aortic occlusion, and we found that the tricuspid valve was dysplastic, the fibrous tissue around the tricuspid valve ring was hyperplastic and the valve leaflets were contracted, which led to severe tricuspid valve stenosis and insufficiency (Fig. 3). All the leaves and subvalvular tissues of the tricuspid valve were removed, and the 17 mm valve was passed (Fig. 4). Sixteen sutures were placed along the valve ring, and a 16 mm Medtronic mechanical mitral valve was implanted (Fig. 5). A 3 mm defect was created in the atrial septum, and then the right atrial incision was sutured. An end-to-side cavopulmonary anastomosis was created on a beating heart (Fig. 6). The open right pulmonary artery was banded to a size of 4 mm. Extracorporeal circulation was gradually removed. After hemostasis, the sternum was closed, and the skin was sutured.

### Treatment after surgery

After the operation, the patient was treated with mechanical ventilation, adrenaline and dopamine, furosemide, nitric oxide, heparin sodium, and cephalosporins. Color ultrasound examination indicated that the fenestrated atrial septal defect was a left to right shunt. Three days after the operation, the ventilator was removed. Treatment with cardiotonics, diuretics and anti-infectives was continued. Bosentan was used to reduce pulmonary vascular resistance, and warfarin was used for anticoagulation. Diuresis was induced with furosemide tablets combined with spironolactone tablets, pulmonary vascular resistance was reduced with bosentan, and anticoagulation was induced with warfarin and continued after discharge. At 1 month, 3 months, and 6 months after the operation, shortness of breath after activities was gradually relieved. Echocardiography showed good tricuspid valve function and patency of the gallbladder anastomosis.

## Discussion

Cardiac valves often grow rapidly in children, but prosthetic valves cannot grow as children grow [7]. In addition, the mitral or tricuspid valve lies in the junction between the left and right cardiac structures, which makes valve replacement very difficult in children. This was evidenced by the poor outcomes of mitral prosthetic valve replacement in children, with a 10-year survival rate of only 33-75% [8]. Therefore, children’s cardiac valve disease is a very difficult problem, especially for children who can only undergo prosthetic valve replacement surgery [9]. Furthermore, because there is less stenosis of the valve ring than that of the normal age, selecting and placing the appropriate type of prosthetic valve to ensure the hemodynamic stability of the patient have always been subjects of debate.

We reported the case of a child treated for severe tricuspid stenosis 7 years after the surgical correction of a complete atrioventricular septal defect. Common causes of long-term valve stenosis after valvuloplasty included valve degeneration, valve thrombosis, and endocarditis. Through intraoperative exploration, due to the difficulty of valvuloplasty for serious tricuspid valve lesions, we could only perform tricuspid mechanical valve replacement. All the tricuspid leaves and subvalvular tissues were removed, and only the 17 mm valve tester could be passed. Only the smaller mechanical mitral valve (16 mm metallic mitral mechanical valve) in China could be implanted. For a 7.5-year-old child, the current mechanical valve might not be a suitable match. Therefore, the opening of his tricuspid mechanical valve was still narrow even after valve replacement. Furthermore, with increasing age, the opening of the tricuspid mechanical valve becomes increasingly narrow, and symptoms of venous obstruction might develop. To solve this problem, we added the Glenn procedure to direct superior vena cava blood flow to the right pulmonary artery. To increase the anterior flow of the superior vena cava to the right pulmonary artery, we performed circumconstriction at the opening of the right pulmonary artery to reduce the flow from the heart to the right pulmonary artery. We also performed an atrial septal stoma to decompress the right cardiac system in the long term. After surgery, the hemodynamics of the patient were stable, and the patient was discharged smoothly.

Although this procedure is beneficial for patients, there are still some limitations of this procedure. Patients who undergo mechanical tricuspid valve replacement need to take warfarin for life, which has risks of bleeding and thrombosis. At the same time, lifelong drug treatment and frequent monitoring of coagulation function affect the quality of life of children. 2. The mechanical valve cannot grow, and the valve will eventually stenose as the child continues to grow, thus requiring reoperation. 3. With the increase in pulmonary resistance, vena cava obstruction or reflux and facial edema may occur in the long term after the Glenn operation.

There were some limitations of my paper. Because it was an operation performed in another hospital, the time was relatively long, and the family did not participate in regular follow-ups so some data were lost. Limitations of our medical tests include the absence of some data.

## Conclusion

Tricuspid mechanical valve replacement is an effective treatment for severe tricuspid stenosis in children and has good short-term results, but the long-term results are still being determined via follow-up. For patients with right hypoplastic ventricles who still have right ventricular function, 1.5 ventricular operations can be performed.

## Data Availability

All data needed to evaluate the conclusions were present in the paper and available in the PubMed database.
